# Extracellular Histones Trigger Disseminated Intravascular Coagulation by Lytic Cell Death

**DOI:** 10.3390/ijms23126800

**Published:** 2022-06-18

**Authors:** Yan Zhang, Congqing Wu, Lan Li, Ankit Pandeya, Guoying Zhang, Jian Cui, Daniel Kirchhofer, Jeremy P. Wood, Susan S. Smyth, Yinan Wei, Zhenyu Li

**Affiliations:** 1 Department of Oncology, the First Affiliated Hospital of Soochow University, Suzhou 215006, China; yzh422@tamu.edu; 2 Saha Cardiovascular Research Center, College of Medicine, University of Kentucky, Lexington, KY 40506, USA; gzh223@tamu.edu (G.Z.); jeremy.wood@uky.edu (J.P.W.); 3 Department of Pharmaceutical Sciences, Irma Lerma Rangel College of Pharmacy, Texas A & M University, College Station, TX 76549, USA; yinan.wei@tamu.edu; 4 Department of Surgery, College of Medicine, University of Kentucky, Lexington, KY 40506, USA; 5 Department of Chemistry, College of Arts and Sciences, University of Kentucky, Lexington, KY 40506, USA; lli269@uky.edu (L.L.); pandeya.ankit@uky.edu (A.P.); jian.cui@uky.edu (J.C.); 6 Department of Early Discovery Biochemistry, Genentech Inc., South San Francisco, CA 94080, USA; dak@gene.com; 7 Internal Medicine, University of Arkansas for Medical Sciences, Little Rock, AR 72205, USA; ssmyth@uams.edu

**Keywords:** platelet, thrombocytopenia, histones, DIC, tissue factor, coagulation, cell death

## Abstract

Histones are cationic nuclear proteins that are essential for the structure and functions of eukaryotic chromatin. However, extracellular histones trigger inflammatory responses and contribute to death in sepsis by unknown mechanisms. We recently reported that inflammasome activation and pyroptosis trigger coagulation activation through a tissue-factor (TF)-dependent mechanism. We used a combination of various deficient mice to elucidate the molecular mechanism of histone-induced coagulation. We showed that histones trigger coagulation activation in vivo, as evidenced by coagulation parameters and fibrin deposition in tissues. However, histone-induced coagulopathy was neither dependent on intracellular inflammasome pathways involving caspase 1/11 and gasdermin D (GSDMD), nor on cell surface receptor TLR2- and TLR4-mediated host immune response, as the deficiency of these genes in mice did not protect against histone-induced coagulopathy. The incubation of histones with macrophages induced lytic cell death and phosphatidylserine (PS) exposure, which is required for TF activity, a key initiator of coagulation. The neutralization of TF diminished the histone-induced coagulation. Our findings revealed lytic cell death as a novel mechanism of histone-induced coagulation activation and thrombosis.

## 1. Introduction

Histones are essential cationic proteins that package DNA in eukaryotic cell nuclei. Extracellular histones, however, have been implicated in several pathophysiological processes [1,2,3]. During the innate immune response, histones released in neutrophil extracellular traps (NETs), along with DNA fibers, play an important role in entrapping and killing bacteria [4]. Extracellular histones are highly toxic independent of NETs. Histones alone, but not NETs, activate coagulation in vitro [5]. It has been reported that extracellular histones contribute to death in sepsis through cell surface pattern recognition receptor TLR2- and TLR4-dependent mechanisms [6,7]. The injection of histones into mice results in a massive prothrombotic response, similar to observations in sepsis, including fibrin and platelet deposition in lung alveoli [7]. Coagulopathy and thrombosis are common complications in COVID-19, especially in patients who do not survive [8,9,10]. Elevated histone H3 has been reported in COVID-19 patients and could contribute to coagulopathy [11].

Several mechanisms have been reported in histone-induced thrombosis. Histones can stimulate platelet aggregation in vitro and the injection of histones into mice results in thrombocytopenia [12,13]. Histones induce the release of the von Willebrand factor from endothelial Weibel–Palade bodies, which also contributes to thrombocytopenia [14]. In addition, histones promote thrombin generation and coagulation activation through platelet-dependent and platelet-independent mechanisms [15,16]. Histones can increase TF activity and enhance thrombin generation in blood monocytes and endothelial cells [17,18]. In this study, we show that histones induce macrophage lysis and phosphatidylserine (PS) exposure, leading to TF activation, which triggers systemic coagulation. Neutralizing TF with a monoclonal antibody protects against histone-induced coagulation. Our findings identify a novel mechanism of thrombosis through histone-induced lytic cell death that is independent of inflammatory receptors and signaling pathways.

## 2. Results and Discussion

### 2.1. Extracellular Histones Trigger Coagulopathy Independent of TLR Receptors and Inflammatory Signaling Pathways

**Figure 1:**
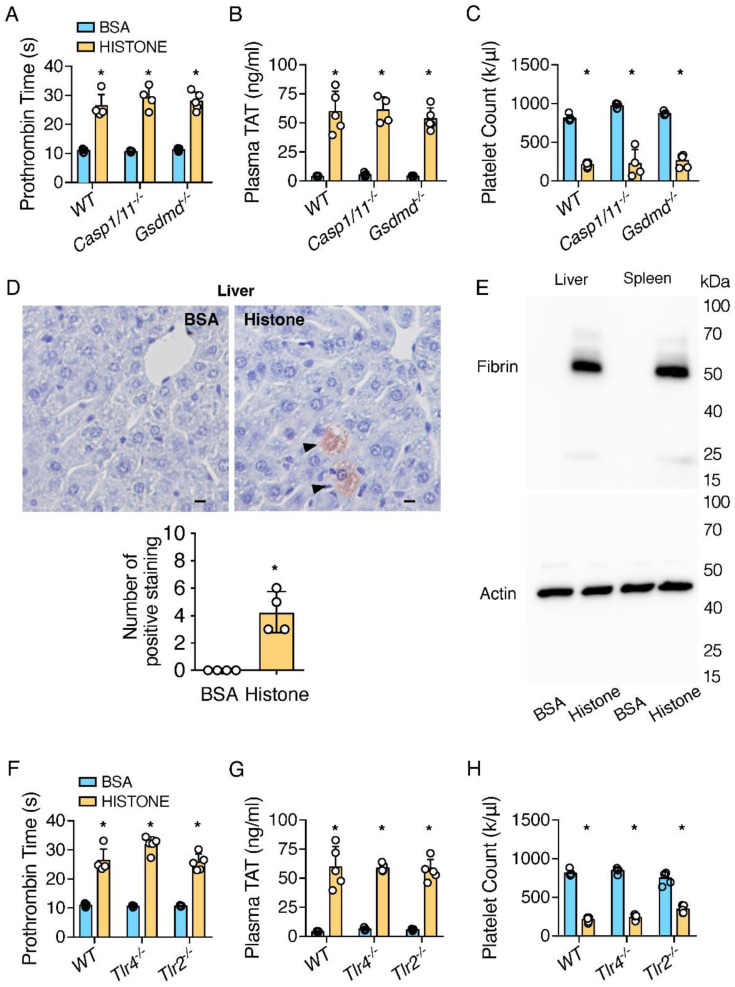
Extracellular histones triggered coagulopathy independent of inflammasome pathways and TLR receptors. (**A**–**C**) C57BL/6J mice (WT), Casp1/11-deficient mice, and GSDMD-deficient mice were injected intravenously with BSA or histones. Blood was collected 60 min after injection. Prothrombin time (**A**), plasma TAT concentrations (**B**), and total platelet count (**C**) were measured. Error bars denote SD; * *p* < 0.05 versus BSA, Mann–Whitney test. (**D**) C57BL/6J mice (WT) were injected intravenously with BSA or histones. After 60 min, the mice were perfused with PBS and then perfusion-fixed with 10% (*v*/*v*) formalin under physiological pressure for 45 min. Liver sections were immunostained with anti-fibrin monoclonal antibody (59D8). Wild-type mice showed fibrin deposition in the liver (arrows). Scale bars denote 10 µm. Numbers of positive staining were quantified on images acquired at 20X or equivalent. Error bars denote SD; * *p* < 0.05 versus BSA, Mann–Whitney test. (**E**) C57BL/6J mice (WT) were injected intravenously with BSA or histones. After 60 min, the mice were euthanized, and their tissues were harvested. Fibrin in the tissue lysates was detected by immunoblot with the anti-fibrin monoclonal antibody 59D8. Data are representative of 3 independent experiments. (**F**–**H**) C57BL/6J mice, TLR4-deficient mice, and TLR2-deficient mice were injected intravenously with BSA or histones. Blood was collected 60 min after injection. Prothrombin time (**F**), plasma TAT concentrations (**G**), and platelet counts (**H**) were measured. Error bars denote SD; * *p* < 0.05 versus BSA, Mann–Whitney test.

Histones have been shown to increase TF activity in cells [17,18]. To investigate whether the injection of histones could directly trigger coagulation activation in vivo, mice were intravenously injected with unfractionated calf thymus histones at 50 mg/kg, a dose comparable to those seen in humans with sepsis/DIC [19]. Patients with DIC exhibit an increased prothrombin time (PT), elevated plasma thrombin anti-thrombin (TAT), and thrombocytopenia [20,21]. Similarly, the injection of histones into mice significantly increased PT (Figure 1A), and plasma TAT was elevated by more than 10-fold in mice that received histones to BSA (Figure 1B). Total platelet counts were decreased by more than 70% (Figure 1C). Since we observed similar coagulation in female mice (Supplementary Figure S1), only male mice were used in subsequent studies. We detected fibrin deposition in the livers of mice treated with histones, using immunostaining and Western blotting (Figure 1D,E). Together, these data demonstrated that histones effectively triggered coagulation activation in vivo.

Our recent work demonstrated that inflammasome activation triggered DIC via pyroptosis [19]. Because histones have been shown to activate inflammasome in vitro [22], we examined whether histone-induced coagulation activation proceeded through inflammasome activation and pyroptosis. To our surprise, neither the deficiency of caspase-1 nor GSDMD had any significant impact on histone-induced coagulopathy measured with PT, plasma TAT, and total platelet counts (Figure 1A–C). This suggested that histone-induced coagulopathy was independent of inflammasome activation.

Since histone-induced organ damage and death in mice involve both TLR2 and TLR4 pathways [6,7], we determined whether the deficiency of TLR2 or TLR4 protected against histone-induced coagulopathy. Increases in PT and plasma TAT concentrations and thrombocytopenia by histone injection were similar in wild-type and TLR2- or TLR4-deficient mice (Figure 1F–H). These data demonstrated that histone-induced coagulopathy did not require TLR2 or TLR4.

### 2.2. Extracellular Histones Lyse Macrophages and Expose PS

**Figure 2:**
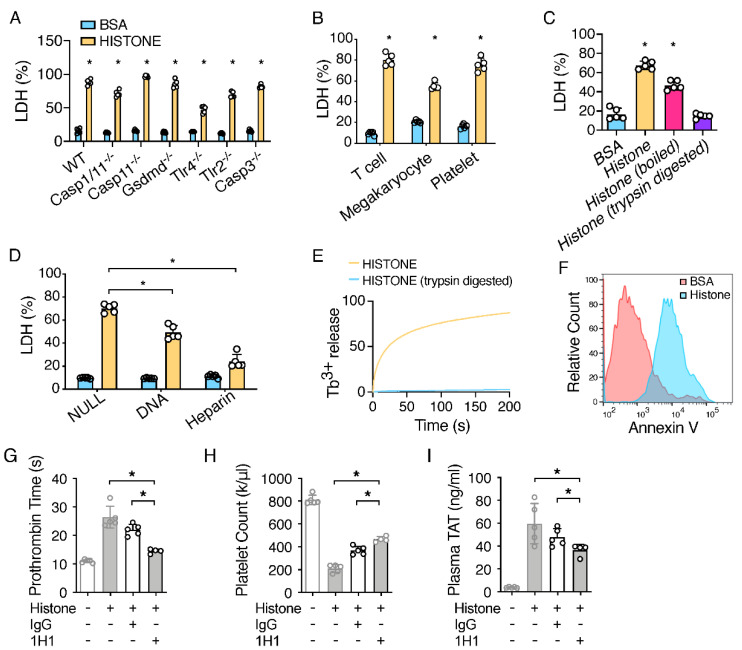
(**A**–**F**) Extracellular histones induced lytic cell death and PS exposure. (**A**) Histones lysed macrophages. BMDMs were isolated from C57BL/6J mice (WT), Casp1/11-deficient mice, Casp11-deficient mice, GSDMD-deficient mice, TLR4-deficient mice, TLR2-deficient mice, and Casp3-deficient mice. Cells were incubated with BSA or histones for 60 min. (**B**) Extracellular histones lysed many other cell types. T cells, megakaryocytes, and platelets were incubated with BSA or histone for 60 min. (**C**) Histones, even when subjected to boiling, lysed macrophages. Histones, boiled histones, and trypsin-digested histones (500 µg/mL) were incubated with BMDM for 60 min. Error bars denote SD; * *p* < 0.05 versus BSA, Mann–Whitney test. (**D**) Both DNA and heparin blocked histone-induced cell lysis. Mouse genomic DNA or heparin were mixed in equal amounts with BSA or histone (500 µg/mL) prior to incubation with BMDM for 60 min. Histone-mediated cell cytotoxicity was determined using an LDH cytotoxicity assay. Error bars denote SD; * *p* < 0.05, Mann–Whitney test. (**E**) Liposome leakage was monitored with terbium (Tb3+) fluorescence after incubation with histones or trypsin-digested histones. (**F**) Histone-induced PS exposure. C57BL/6J mice (WT) BMDMs were treated with BSA or histones for 15 min, and then stained with Alexa 488-labeled Annexin-V and analyzed with flow cytometry. (**G**–**I**) TF neutralization protected against histone-induced coagulopathy. C57BL/6J mice (WT) were injected intravenously with a rat IgG or rat anti-mouse TF-neutralizing antibody 1H1 (8 mg/kg). After 2 h, the mice were injected intravenously with histones. Blood was collected 60 min after histone injection. Prothrombin time (**G**), plasma TAT concentrations (**H**), and total platelet count (**I**) after histone injection were measured. Error bars denote SD; * *p* < 0.05, Mann–Whitney test.

We have recently shown that TF from pyroptotic macrophages plays an important role in sepsis-associated DIC [23]. Thus, we investigated whether histones could also induce TF activation in macrophages. The incubation of mouse bone-marrow-derived macrophages (BMDMs) with histones resulted in cell death within one hour in a dose-dependent manner (Figure 2A; Supplementary Figure S2). All four histone fractions, H2A, H2B, H3, and H4, contributed to histone-induced cell death (Supplementary Figure S3). Surprisingly, the deficiency of caspase-1/11 or GSDMD failed to protect against histone-induced cell death (Figure 2A). These results indicated that histone-induced cell death proceeded through an inflammasome activation/pyroptosis-independent mechanism. A deficiency in caspase-3 did not protect against histone-induced cell death (Figure 2A), suggesting that histone-induced cell death was not due to apoptosis. In further agreement with our in vivo observations (Figure 1A–C), neither TLR2 nor TLR4 deficiency prevented histone-induced cell death (Figure 2A).

Histones are positively charged proteins, which may allow for high-affinity binding with the negatively charged cell membrane, which could lead to the lytic cell death of smooth muscle cells through a non-programmed cell death mechanism [24]. In agreement with this, we found that histones induced cell death in different types of cells, including platelets, megakaryocytes, and T lymphocytes (Figure 2B). To investigate the mechanism of cell killing, we incubated BMDMs with histones that were either boiled, to induce denaturation, or proteolytically digested with trypsin. Boiled histones, but not trypsin-digested histones, induced cell death in BMDMs, similar to untreated histones (Figure 2C). To test whether histones disrupted cell integrity via its positive charges, histones were mixed with negatively charged DNA or heparin and then co-incubated with macrophages. The pre-incubation of histones with DNA or heparin protected against the death of macrophages (Figure 2D; Supplementary Figure S4), which was consistent with previous studies showing that histones, as part of the nucleosome complex, were not cytotoxic [25], and that heparin prevented histone-induced toxicity in vivo [13]. It appears that DNA is less effective than heparin in preventing cell death by histones. This may be because DNA is not as efficient as heparin at neutralizing histones. However, we could not exclude the possibility that a positive-charge-independent mechanism also contributes to cell death by histones. We further observed that histones disrupted the membranes of liposomes pre-packaged with Tb3+, as indicated by the time-dependent increase in Tb3+ release (Figure 2E). The leakage of the cell membrane led to PS exposure on the outer membrane leaflet, which has been shown to be required for TF activity, the key initiator of coagulation. In agreement with this, the incubation of BMDMs with histones did induce PS exposure, as shown by Annexin V staining (Figure 2F).

### 2.3. TF Neutralization Protects against Extracellular Histone-Induced Coagulopathy

To determine whether TF is required for histone-induced coagulopathy, we utilized an inhibitory rat anti-mouse TF antibody, 1H1, to block TF activity [26]. Indeed, mice that were administered 1H1, but not a control IgG, were protected from histone-induced coagulopathy (Figure 2G–I), suggesting that TF plays an important role in histone-induced coagulopathy. However, pre-treatment with 1H1 did not fully rescue thrombocytopenia induced by histones (Figure 1H and Figure 2I). These data suggest that TF-independent mechanisms may also contribute to histone-induced thrombocytopenia and coagulation activation. In this regard, it has been shown that histones can activate platelets through integrin α2bβ3 [3,12]. In addition, histones can induce coagulation activation through NET formation [27].

The injection of histones triggered coagulation and DIC. Our results demonstrated that TF released from lytic cells, likely from macrophages, plays an important role in histone-induced coagulation activation and thrombosis. We also identified that histone induces cell death through the disruption of membrane integrity via its positive charges. These findings did not exclude the possibility that other mechanisms may also be involved in histone-induced coagulopathy. Except through triggering NET formation [27], histones facilitated the FXa cleavage of prothrombin to release active thrombin through directly binding to prothrombin fragment 1 and 2 [28].

## 3. Materials and Methods

### 3.1. Mice

C57BL/6J, Casp1/11^-/-^, Casp11^-/-^, Tlr4^-/-^, Tlr2^-/-^, Gsdmd^-/-^, and Casp3^-/-^ mice were housed in the University of Kentucky Animal Care Facility, following institutional and National Institutes of Health guidelines after approval by the Institutional Animal Care and Use Committee (A3336-01). Since coagulation was observed similarly in female mice, only male mice were used in all subsequent studies. Male mice at 8–12 weeks were used in all experiments, unless stated otherwise.

### 3.2. In Vivo Challenges

Histones (Worthington Biochemical Corp., Lakewood, NJ, USA cat#LS002546/lot#33P14617) were administered at 50 mg/kg via retro-orbital injection.

### 3.3. Pharmacological TF Inhibition

Rat IgG (Sigma-Aldrich Inc., St. Louis, MO, USA Cat#I4131) or 1H1 anti-TF antibody (Genentech, San-Francisco, CA, USA) at 8 mg/kg was given via retro-orbital injection 2 h prior to histone challenge.

### 3.4. Measurement of Coagulation

Blood was collected from tribromoethanol-anaesthetized mice by cardiac puncture with a 23-gauge needle attached to a syringe pre-filled with 3.8% (*w*/*v*) trisodium citrate as an anticoagulant (final ratio at 1:10). Blood was collected 60 min after histone injection as inflammasome activation triggered blood coagulation as early as 60 min after histone injection. Blood was centrifuged at 1500× *g* for 15 min at 4 °C to obtain plasma. Prothrombin time (PT), plasma TAT concentrations, and platelet counts were measured as described previously [19]. Briefly, PT was determined with Thromboplastin-D (Thermo Scientific Pacific Hemostasis, Waltham, MA, USA Cat#100357/lot965299) in a manual setting according to manufacturer’s instructions, using a CHRONO-LOG #367 plastic cuvette. Plasma TAT levels were determined using an ELISA kit (Abcam, Boston, MA, USA Cat#ab137994) at a 1:50 dilution. Total platelet counts were acquired on a ProCyte Dx Hematology Analyzer (IDEXX).

### 3.5. Tissue Preparation and Immunohistochemistry

Mice were perfused via both right and left ventricles with PBS and then perfusion-fixed with 10% (*v*/*v*) formalin under physiological pressure for 30–45 min. Tissues were collected and embedded in paraffin, then sectioned serially at 5 µm. An anti-fibrin antibody 59D8 at 4 µg/mL was used for staining fibrin deposition, with a biotinylated goat anti-mouse IgG at a 1:200 dilution used as a secondary antibody for developing positive staining.

### 3.6. BMDM Cultures

BMDMs were isolated and seeded into 12-well cell culture plates at a density of 1 × 10^6^ cells/well in 1 mL of RPMI-1640 medium containing 15% (*v*/*v*) L929-cell-conditioned medium (LCM) [29,30]. BMDMs were allowed to settle overnight and were refreshed with 1 mL of Opti-MEM (Thermo, Waltham, MA, USA Cat#31985-070) before BSA or histones were added (500 µg/mL).

### 3.7. Detection of Fibrin in Tissues by Western Blot

Frozen tissues were homogenized in 10 volumes (mg:µL) of T-PER tissue protein extraction reagent (Thermo, Cat#78510) containing cocktail inhibitor (Sigma, Cat#P8340) and PMSF. After centrifugation at 10,000× *g* for 10 min, the supernatant was collected for beta-actin detection. Pellets were homogenized in 3 M urea and vortexed for 2 h at 37 °C. After centrifugation at 14,000× *g* for 15 min, resulting pellets were suspended in SDS-PAGE sample buffer and vortexed at 65 °C for 30 min. The samples were analyzed with SDS-PAGE on 4~15% gradient gels in reduced condition and immunoblotted using 59D8 at 0.5 µg/mL.

### 3.8. Liposome Leakage Assay

A liposome leakage assay was conducted as described [20]. Briefly, a SX20 LED stopped-flow spectrometer (Applied Photophysics Ltd., Beverly, MA, USA) equipped with a 280 nm LED light and a 400 nm cutoff filter was used to monitor the increase in fluorescence upon liposome leakage. To obtain the percentage leakage data, TX-100 0.5% (*w*/*v*) was used to dissolve the liposome and completely release Tb3+. The increase in intensity upon mixing was determined and used as the 100% leakage value F0. The fluorescence intensity was normalized to F0 to calculate the percentage of leakage.

### 3.9. Cytotoxicity Assays

BMDMs cell death was determined using an LDH Cytotoxicity Detection Kit (Promega, Madison, WI, USA Cat#G1780). Briefly, 50 µL of assay buffer was added to each well containing 50 µL cell culture (1 × 10^5^ cells) in a 96-well plate. After incubation for 5–10 min, the absorbance was recorded at 490 nm.

### 3.10. Flow Cytometry

Annexin V-FITC Apoptosis Detection kit (Thermo, Cat#V13241) was used to detect the PS exposure of cells in the study. Data were acquired on a CytoFlex (Beckman Coulter, Brea, CA, USA) and analyzed with FlowJo v10.07.

### 3.11. Statistical Analysis

Data are represented as mean ± SD. The Mann–Whitney test was performed in Prism 8 to compare two-group data. *p* < 0.05 was considered statistically significant.

## 4. Conclusions

In sepsis, many mechanisms contribute to DIC. However, an antibody to histones reduced the mortality of mice in lipopolysaccharide (LPS) and cecal ligation and puncture models of sepsis [7], suggesting that histone-induced coagulation activation is important in sepsis. Therefore, treatments that protect against histone-induced cell death may potentially increase the survival of septic patients.

## Data Availability

Data will be made available by the corresponding authors upon request.

## Supplementary Materials

The following are available online at https://www.mdpi.com/article/10.3390/ijms23126800/s1.
