# Supplementary material for: Extracellular Histones Trigger Disseminated Intravascular Coagulation by Lytic Cell Death
Source: Int J Mol Sci. 2022 Jun 18;23(12):6800. doi: 10.3390/ijms23126800 (PMC9224270; doi:10.3390/ijms23126800)
Supplement: Supplementary file 1 [file ijms-23-06800-s001.zip › ijms-1695622-supplementary.pdf]

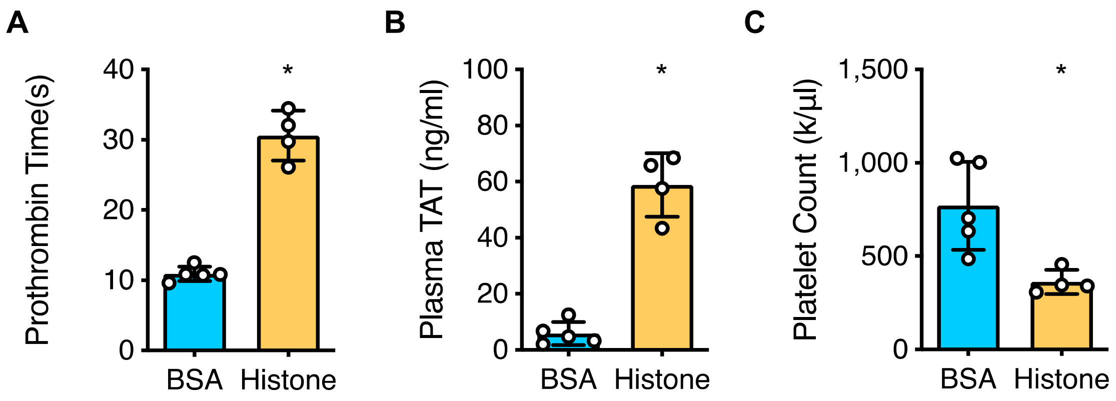

Figure S1. Extracellular histones trigger coagulopathy in female mice (**A-C**) Female C57BL/6J mice were injected intravenously with BSA or Histones. Blood was collected 60 minutes after injection. Prothrombin time (**A**), plasma TAT concentrations (**B**), and total platelet count (**C**) were measured. Error bars denote SD; * $P < 0.05$ versus BSA, Mann-Whitney test.

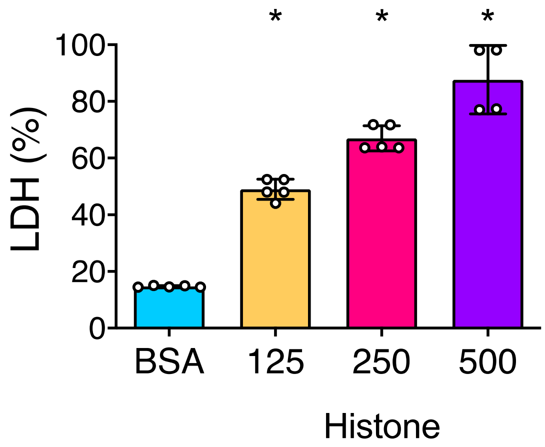

Figure S2. Histones induce lytic cell death in a dose-dependent manner. BMDMs were isolated from C57BL/6J mice. Cells were incubated with BSA (500 μg/mL) or histones at 125, 250, 500 μg/mL for 60min. Error bars denote SD; * $P < 0.05$ versus BSA, Mann-Whitney test.

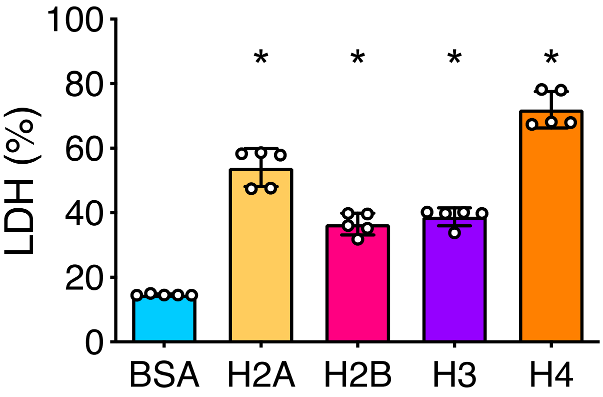

Figure S3. Human recombinant histone H2A, H2B, H3, and H4 induce lytic cell death. BMDMs were isolated from C57BL/6J mice. Cells were incubated with BSA or human recombinant histones (125 μg/mL, obtained from New England Biolabs) for 60min. Error bars denote SD; * $P < 0.05$ versus BSA, Mann-Whitney test.

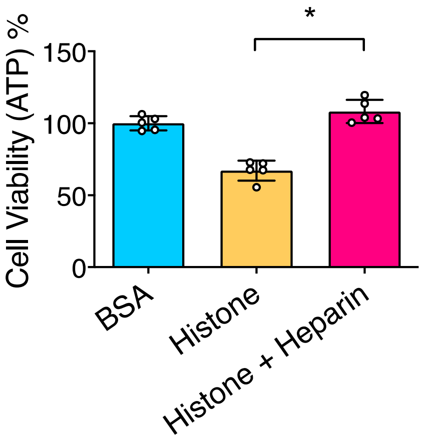

Figure S4. Heparin inhibits histone-induced lytic cell death when incubated in mouse plasma. BMDMs were isolated from C57BL/6J mice. Cells were incubated with BSA or histones for 60min in mouse plasma (EDTA-treated) instead of cell culture medium. Heparin was mixed at equal amount with histone (500 μg/mL) prior to incubation with BMDM for 60 min. Cell viability (ATP) was measured by CellTiter-Glo Luminescent Cell Viability Assay. Error bars denote SD; * $P < 0.05$ versus Histone, Mann-Whitney test.
